# AAV6 Vexosomes Mediate Robust Suicide Gene Delivery in a Murine Model of Hepatocellular Carcinoma

**DOI:** 10.1016/j.omtm.2020.03.006

**Published:** 2020-03-13

**Authors:** Nusrat Khan, Shubham Maurya, Sridhar Bammidi, Giridhara R. Jayandharan

**Affiliations:** 1 Department of Biological Sciences and Bioengineering, Indian Institute of Technology, Kanpur 208016, Uttar Pradesh, India

**Keywords:** vexosome, exosome, AAV6, suicide gene delivery

## Abstract

During recombinant Adeno-associated virus (AAV) production, a proportionately large amount of vectors is released in the culture supernatant, which is often discarded. It has been shown that these vectors often associate with vesiculated structures, such as exosomes. Exosome-associated AAV (vexosomes) represent an additional gene-delivery platform. The efficiency of such vexosomes in suicide gene therapy is unexplored. In the present study, we have generated AAV serotype 6 vexosomes containing an inducible caspase 9 (*iCasp9*) suicide gene by a differential ultracentrifugation-based protocol. We further tested the cytotoxic potential of these vexosomes in a human hepatocellular carcinoma (HCC) model *in vitro* and *in vivo*. The AAV6-*iCasp9* containing vexosomes, when primed with a pro-drug (AP20187), demonstrated a significant loss in cell viability (57% ± 8% versus 100% ± 4.8%, p < 0.001) in comparison to mock-treated Huh7 cells. An intratumoral administration of AAV6-*iCasp9* vexosomes and AP20187 in a murine xenograft model revealed a 2.3-fold increase in tumor regression in comparison to untreated animals. These findings were further corroborated by histological analysis and apoptosis assays. In conclusion, our data demonstrate the therapeutic potential of AAV6 vexosomes in a xenotransplantation model of HCC. Furthermore, the simplicity in production and isolation of vexosomes should further facilitate its application in other malignancies.

## Graphical Abstract

**Figure:**
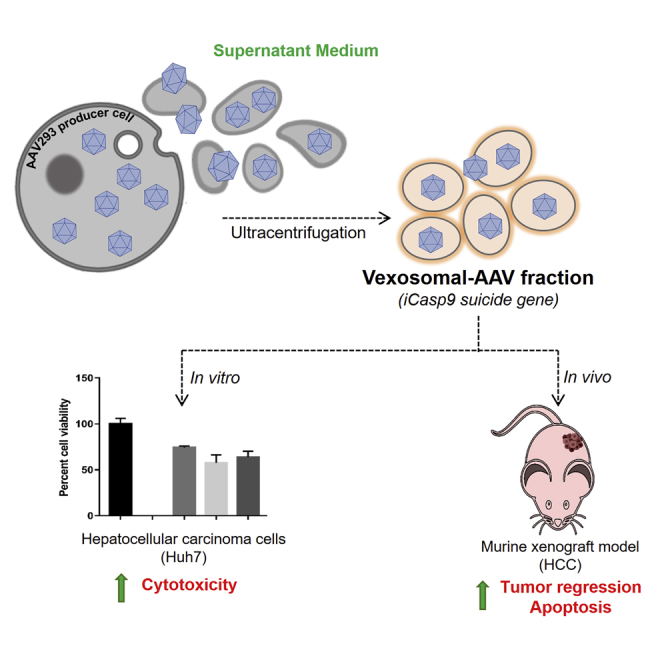

## Teaser

Exosome-associated Adeno-associated virus (AAV) vectors (vexosomes) have gained attention as a potent gene-delivery platform. Khan et al. demonstrate that AAV6 serotype-based vexosomes, containing an inducible caspase 9 gene, are cytotoxic in the presence of a pro-drug AP20187 and have a significant therapeutic potential for suicide gene therapy in hepatocellular carcinoma.

## Introduction

Adeno-associated virus (AAV) vectors have been gaining importance as an efficient delivery system for *in vivo* gene transfer, owing to their long-term gene expression and broad tissue tropism.^1^^,^^2^ They have exhibited an excellent safety profile in clinical trials for hemophilia^3^ and Leber congenital amaurosis (LCA).^4^^,^^5^ However, the requirement of high vector doses (10^12^ vector genomes [vgs] per kilogram), particularly in the context of systemic gene transfer into humans,^3^^,^^6^ necessitates large-scale production of these vectors, thus limiting their widespread use. The arduous and intensive multistep purification protocol is one of the major factor contributing to the cost of this promising mode of gene therapy.^7^, ^8^, ^9^ Thus, further improvements to increase the yield and simplify the downstream purification process are pertinent to address the future needs in clinical application.

The standard method for generating AAV vectors involves the use of a producer cell line AAV293.^10^ During vector production, assembled AAV vector particles that accumulate inside the producer cells are harvested by cell lysis to release AAV particles, followed by different purification steps, such as ultracentrifugation and/or affinity-based purification methods.^11^^,^^12^ Recently, it has been reported that during vector production, a fraction of AAV vectors associated with microvesicles/endovesicles (exosome [exo]-associated AAVs or vexosomes) are naturally released into the supernatant fraction of the cell-culture media.^13^^,^^14^ These exo-AAV vectors seem to perform better than the conventionally purified AAV vectors^15^^,^^16^ in the setting of gene transfer to the retina,^15^ the nervous system,^16^ and the inner ear.^17^ Several studies have also demonstrated that exo-vectors not only have higher transduction efficiency but are also resistant to neutralizing antibodies.^17^^,^^18^ The latter feature may be of importance, particularly for therapeutic applications *in vivo* where endogenous anti-AAV antibodies often compromise the therapeutic efficacy of gene delivery.^13^

Whereas the efficiency of exo-AAV vectors during production of AAV serotypes, such as 1, 2, 5, 6, 7, 8, and 9, has been described^13^ and tested for replacement gene therapy of disorders, such as hemophilia,^17^ Leber congenital amaurosis,^19^ and hearing loss,^18^ its efficiency for suicide gene therapy is not known. We have recently demonstrated the potential of a AAV2-mediated inducible caspase 9 (*iCasp9*) gene delivery in a hepatocellular carcinoma (HCC) model.^20^ In the current study, we have evaluated the potential of exosome-associated AAV6 vectors (exo-AAV6 or AAV6 vexosomes) for their ability to deliver a suicide gene efficiently in an *in vitro* hepatic cancer model (Huh7 cells), as well as its therapeutic potential in the xenograft mice model of HCC.

## Results

### Size Distribution and Marker-Based Assessment of Vexosomes

**Figure 1:**
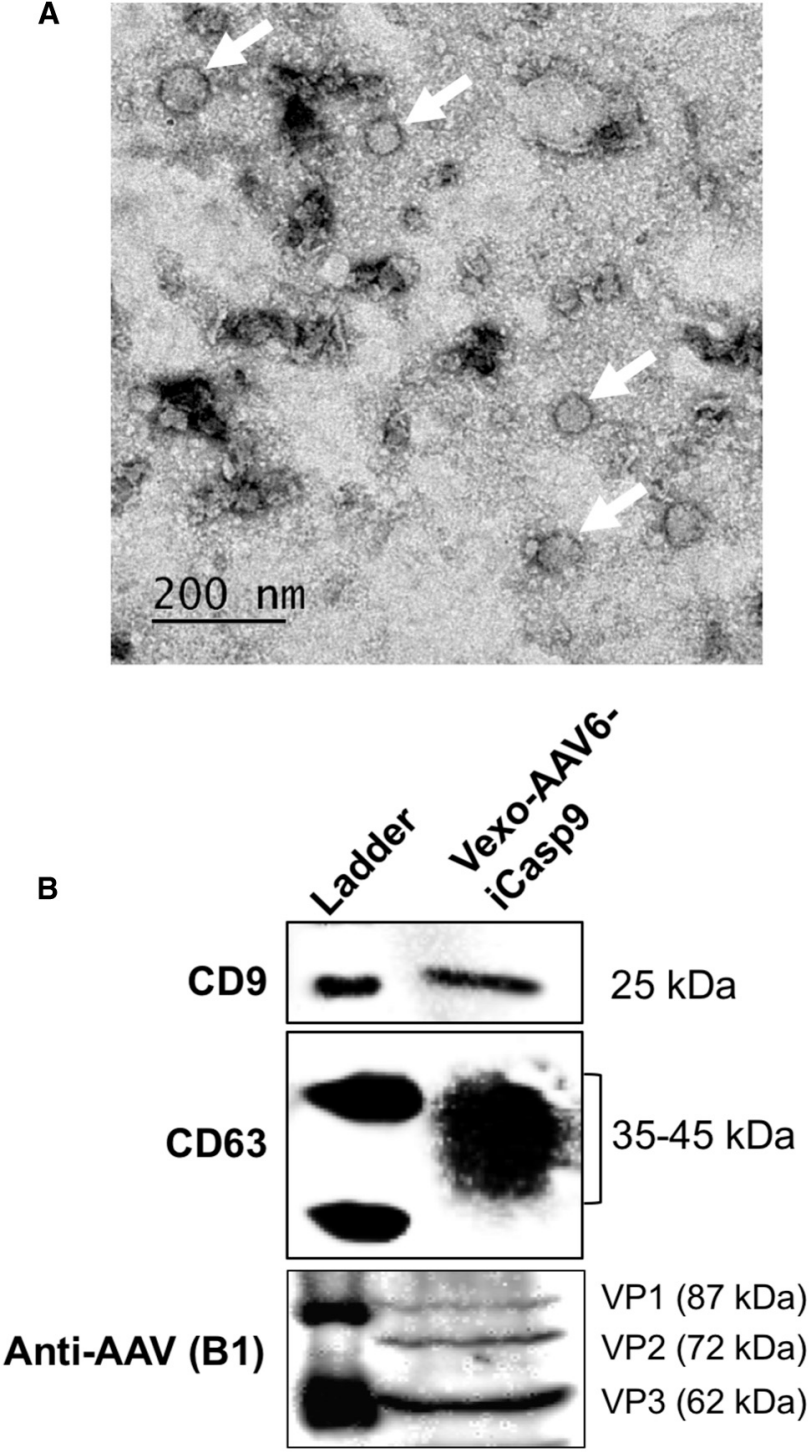
Characterization of Vexosomes Pelleted from AAV Vector-Producing AAV293 Cell-Culture Media (A) Transmission electron microscopy- The representative image shows the presence of vexosomes of varying size. Arrows show the vexosomal membrane. Scale bar, 200 nm. (B) Immunoblotting- total cell proteins were harvested and resolved on a 10% SDS-PAGE gel. Western blotting was performed to detect specific exosomal marker proteins (CD9 and CD63) or AAV capsid proteins (VP1–3). The immune-reactive bands were detected by chemiluminescence imaging. Representative images from three biological replicates are shown.

In the initial set of investigations, cell-culture media from AAV6-producing AAV293 cells were subjected to ultracentrifugation, and a morphological characterization of the vexosome pellet was performed by transmission electron microscopy (TEM). Vexosomes isolated using the classical ultracentrifugation method showed a size distribution from 30 to 130 nm, with a median size of 50 nm (Figure 1A). These data are within the accepted size range for exosomes (<150 nm).^21^ Further quantification of the samples with an Exocet Exosome Quantitation Kit (System Biosciences, Mountain View, CA) for their exosomal yield showed that ∼5 × 10^7^ vexosome particles were present per microliter of sample analyzed (data not shown).

We further characterized the vexosomes by immunoblotting for specific marker proteins. CD9 and CD63 are a set of specific tetraspanin markers that are enriched in exosomes.^22^ We observed that the exosomal fractions were positive for CD9 as well as CD63 markers (Figure 1B), as described earlier.^17^^,^^23^ Similarly, vexosomal lysates also demonstrated the presence of AAV capsid proteins (viral capsid protein [VP]1–3) (Figure 1B), highlighting the presence of AAV vectors in the vexosomal isolates. However, further immunolabeling of both the exosome membrane and AAV capsid and their detailed biophysical analysis may be necessary to quantitate the vector particles within the exosomes.

### Distribution of AAV6 Vectors in Cell Lysates and Culture Media

It has been previously shown that the amount of exosome released into the supernatant media is serotype^13^^,^^24^ and time dependent.^25^^,^^26^ In order to determine the fraction of AAV6 vectors released into the supernatant media, cells and spent culture media were harvested on day 3, post-transfection (as described in Materials and Methods). DNase-resistant vector genomes in both cells and medium were assessed using the quantitative real-time PCR-based assay. Quantification of the AAV genome titers (Table 1) revealed that the overall yield of AAV vector harvested from the culture medium in this experiment was 1.22 × 10^11^ vgs. The vector yield obtained from vexosomes in culture medium was one-half of the yield to naked AAV6 vectors isolated, as described previously.^13^^,^^17^ It is possible that some of this loss could be attributed to the challenges associated with purification of vexosomes from a large volume of spent media.

**Table 1:** Comparison of Conventional and Vexosome-Associated AAV6 Vectors Isolated for This Study

	Conventional AAV6	Vexo-AAV6
Source	cell lysate	conditioned media
Titer (vg/mL)	1.1 × 10^12^	4.9 × 10^11^
Isolation	complex and time consuming	simple and quick
Method of isolation	iodixanol-gradient ultracentrifugation/column purification	ultracentrifugation
Composition	highly pure AAV vector	Vexo-AAV/associated host cellular proteins

### Vexosome (Vexo)-AAV6-*iCasp9* Exhibits Enhanced Cytotoxicity *In Vitro*

**Figure 2:**
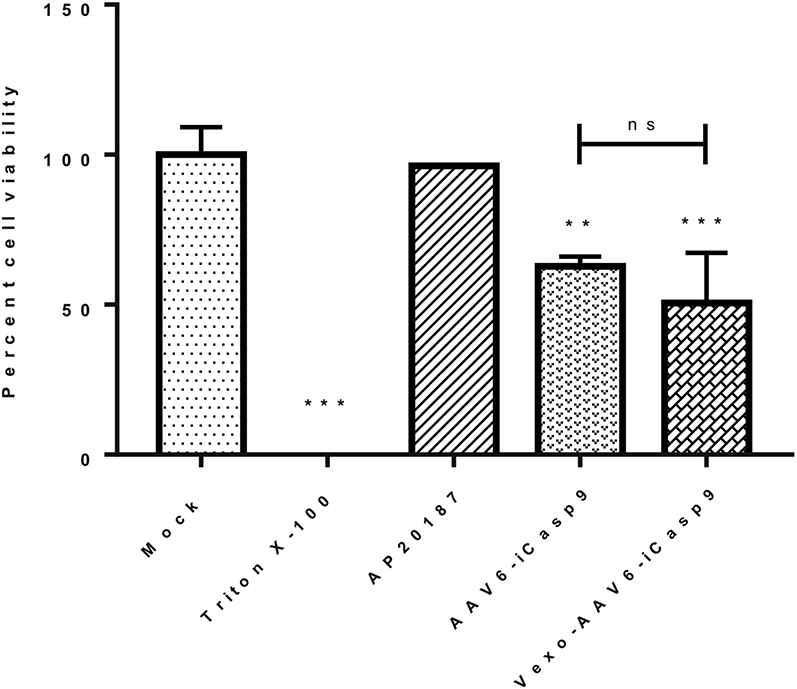
Vexosomes Are Cytotoxic to Hepatocellular Carcinoma, Huh7 Cells *In Vitro* Huh7 cells were infected with either AAV6 or Vexo-AAV6-*iCasp9* vectors, at a multiplicity of infection (MOI) of 5 × 10^4^ vgs/cell, followed by treatment with AP20187. The viability of Huh7 cells treated with a combination of vector and dimerizer drug, after 72 h of vector infection, was determined using an ATP assay (Promega) and depicted in comparison to mock-treated cells. Triton X-100, positive control; AP20187, drug-only control. Data are mean ± SD from two independent experiments (n = 3 replicates each condition per experiment, ∗∗p < 0.01, ∗∗∗p < 0.001 in comparison to mock-treated cells). ns, not significant.

To study the *in vitro* transduction efficiency of AAV6 vectors harvested from two distinct sources, Vexo-AAV6 and conventional AAV6 vectors were generated with the *iCasp9* suicide gene. Huh7 cells were infected with either Vexo-AAV6 or conventional AAV6 vectors at a MOI of 5 × 10^4^, and their cytotoxic effect was measured with an ATP based apoptosis assay. Our outcome data showed that the cell viability in the case of Vexo-AAV6-*iCasp9*-treated cells was substantially reduced to ∼57% when compared with mock-treated cells (57% versus 100%, p < 0.001) (Figure 2). Conventional AAV6-*iCasp9* vectors also demonstrated similar cytotoxicity on Huh7 cells (cell viability 63% versus 100% in mock group, p < 0.001). The functional equivalence seen between the naked and vexosomal vectors here has been previously reported for other serotypes, such as AAV2.^13^

### Vexo-AAV6 Vectors Mediate Significant Tumor Regression in a HCC Xenotransplantation Model

**Figure 3:**
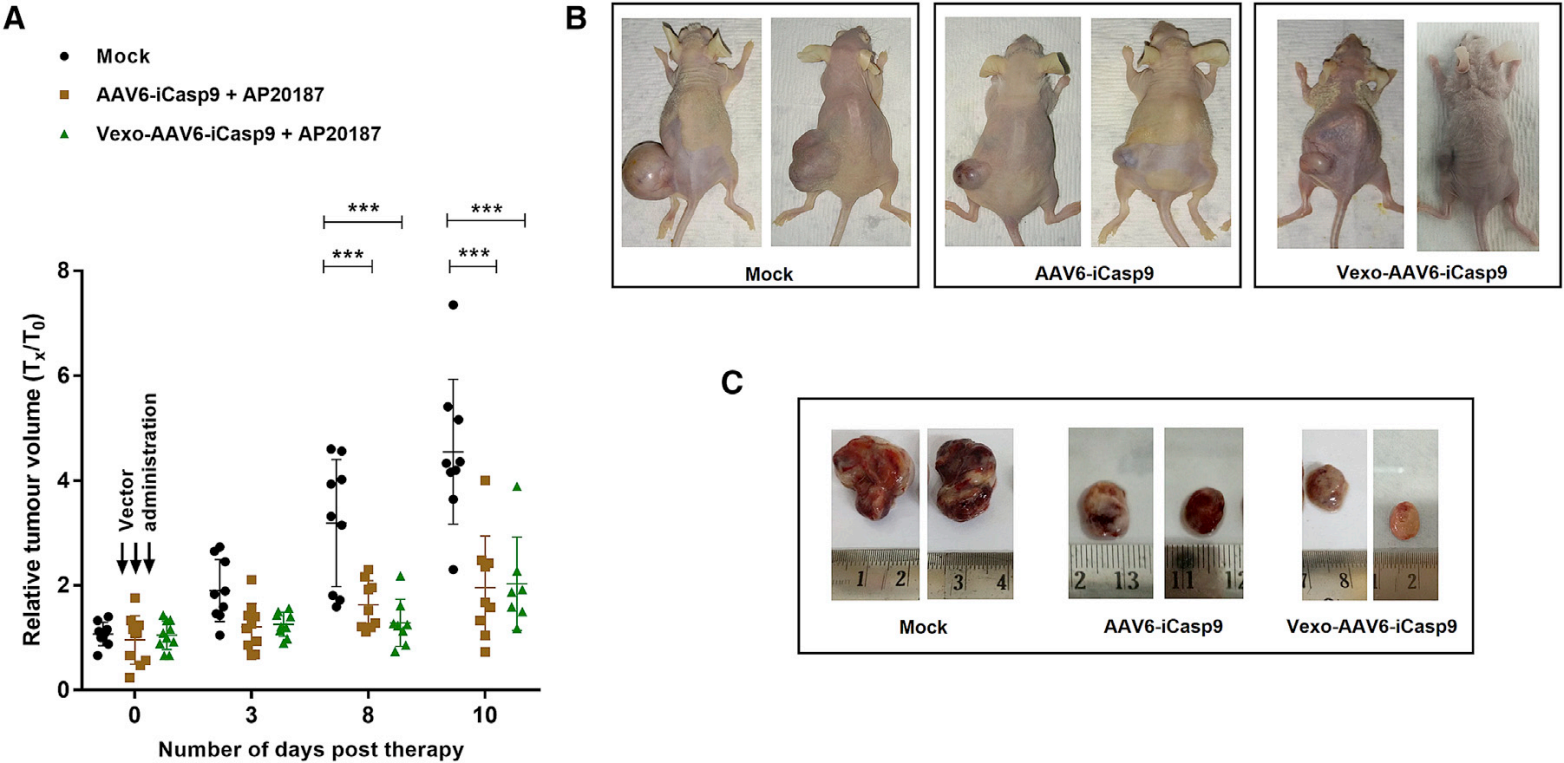
Vexo-AAV6-*iCasp9* Vectors Inhibit Tumor Growth in a Huh7 Cell Xenograft Model (A) HCC tumor-bearing mice were injected with PBS (Mock) intratumorally or with AAV6-*iCasp9* or Vexo-associated AAV6-*iCasp9* vectors (2 × 10^10^ vgs/animal). Animals from both the AAV6-*iCasp9* and Vexo-AAV6-*iCasp9* group demonstrated significant tumor regression when compared to mock-injected animals (data are mean ± SD, n = 7–10 per group, ∗∗∗p < 0.001). (B and C) Representative images of animals (B) and their tumors after enucleation (C).

To validate the therapeutic utility of Vexo-AAV vectors, we utilized a xenotransplantation model of HCC, as described in Materials and Methods. About 15 to 20 days after Huh7 cell transplantation, mice developed tumor nodules. At this time point, we injected the tumor-bearing mice with an equivalent vector dose (2 × 10^10^ vg) of AAV6-*iCasp9* or Vexo-AAV6-*iCasp9* vectors. The control group of mice received PBS injections. A day later, we administered the dimerizer drug (AP20187, 1 mg/kg body weight) intraperitoneally, as described earlier.^27^ The experimental animals were then followed up for ∼10 days to limit the tumor burden in untreated mice, as described earlier.^28^ Some animals in treatment group were also lost during follow-up period, thus possibly requiring further dose-optimization. We observed a significant regression pattern in the tumor volumes of animals that were challenged with Vexo-AAV6-*iCasp9*- but not in the animals of the mock-treated group (Figure 3). The rate of tumor inhibition was ∼2.4-fold for the Vexo-AAV6-*iCasp9* group on day 8, whereas it was ∼2-fold for the AAV6-*iCasp9*-administered group. The regression rate further increased to ∼2.35-fold in the case of AAV6-*iCasp9*, whereas it was ∼2.3-fold for the Vexo-AAV6-*iCasp9* group by day 10 (Figure 3A). These data demonstrate that Vexo-AAV6 vectors are therapeutic in a xenotransplantation model of HCC and appear to be functionally equivalent to conventional AAV6 vectors in terms of suicide gene transfer efficiency both *in vitro* and *in vivo*.

### Administration of Vexo-AAV6 Enhances Apoptosis in the Recipient Tumor Tissue

**Figure 4:**
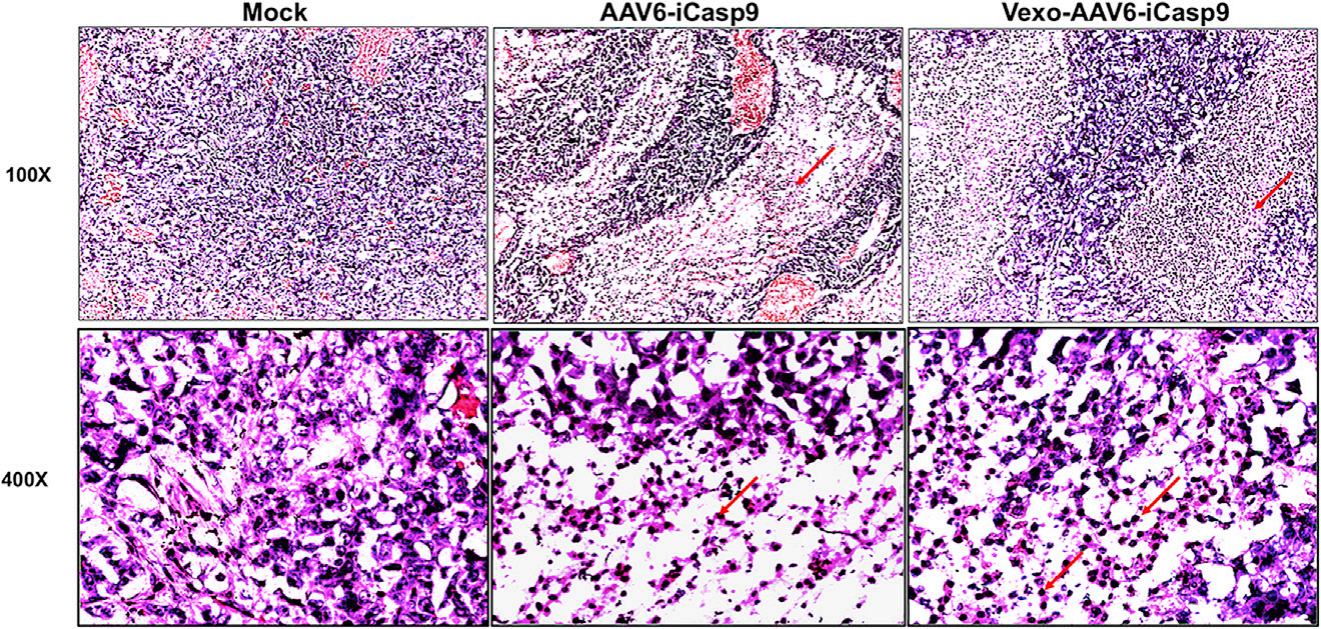
Histological Analysis of Huh7 Xenograft Tumor Tissue Excised tumors were sectioned and stained with hematoxylin and eosin at the end of the experiment. Representative images are shown. A notable pyknosis (red arrow) and apoptosis are seen with Vexo-AAV6-*iCasp9* vector + AP20187-administered animals (magnifications: top, 100×; bottom, 400×).

Tumor-bearing animals from mock or treated groups were humanely euthanized at the end of the experiment, and the subcutaneous tumors were harvested 10 days after gene transfer. For morphological characterization in tissue sections, we performed hematoxylin and eosin staining. Animals from the mock-administered group showed a higher number of mitotically active, large proliferating cells (Figure 4). In the case of tissue sections obtained from the Vexo-AAV6-*iCasp9* vector-treated group, where a significant tumor regression was seen, a significant number of small, condensed apoptotic cells with a pyknosis of their nuclei were observed (Figure 4).

**Figure 5:**
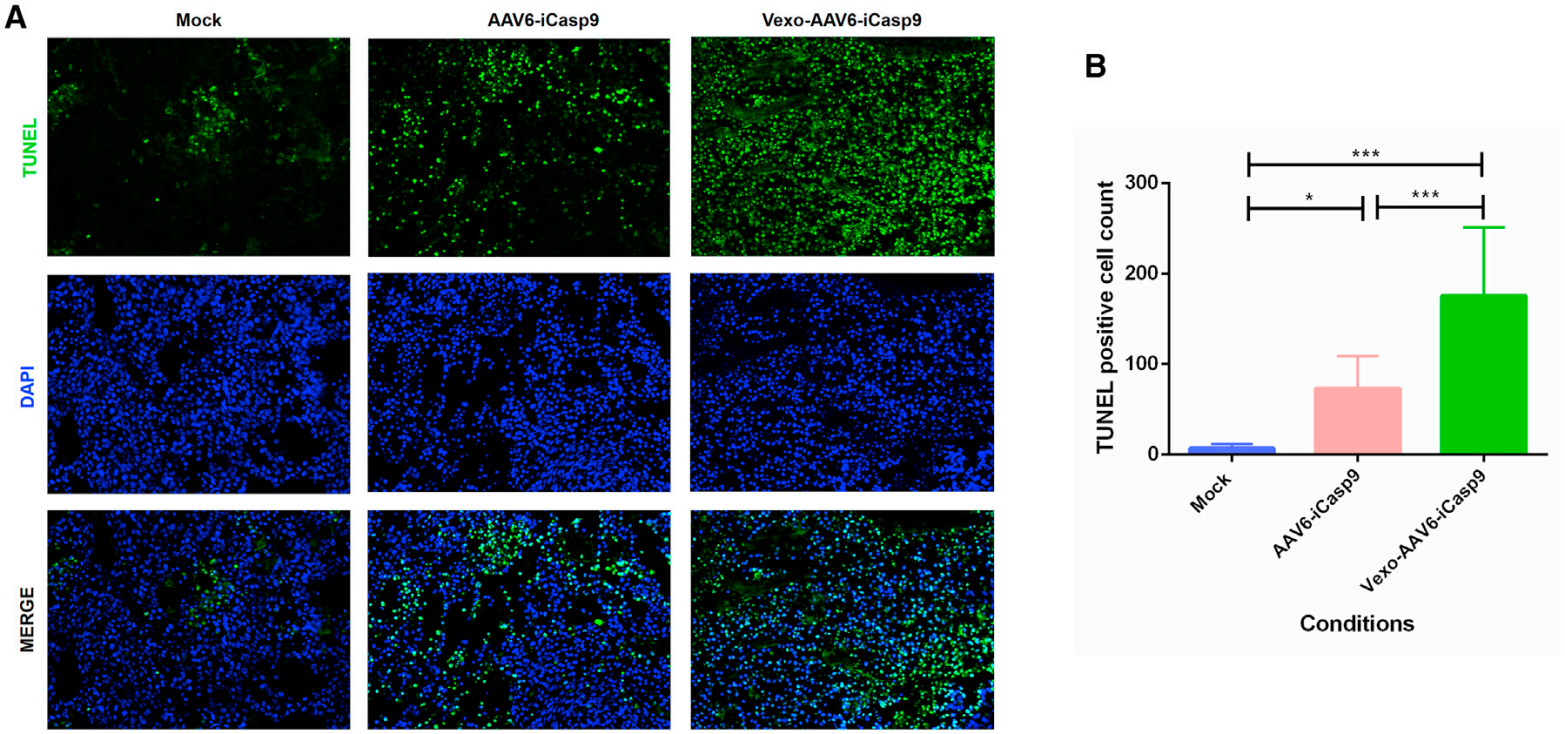
TUNEL Staining in Huh7 Xenograft Tumor Tissue (A) Representative images showing massive apoptosis within the tumors harvested from the Vexo-AAV6-treated group as compared to the mock group (magnification, 200×). Nuclei stained with the TUNEL assay are green. Sections were counterstained with DAPI. (B) Data from (A) (9 sections per condition) were quantified by ImageJ analysis and expressed as mean ± SD (∗p < 0.05, ∗∗∗p < 0.001).

In order to confirm these observations further, a terminal deoxynucleotidyl transferase-mediated deoxyuridine 5-triphosphate (dUTP) nick-end labeling (TUNEL) assay was performed.^29^, ^30^, ^31^ The tumor sections from the Vexo-AAV6-*iCasp9*- or standard AAV6-*iCasp9*-treated group showed marked presence of proapoptotic cells (green color) in comparison to sections prepared from the control animal group (Figure 5A). A quantitative assessment of the extent of DNA damage by ImageJ analysis (Figure 5B) revealed a significant proportion of TUNEL-positive cells in the case of the Vexo-AAV6-*iCasp9* vector-administered group (175 ± 75) in comparison to either the conventional AAV vector-administered (72 ± 35, p < 0.001) or the mock-administered group (7 ± 4, p < 0.001). These data also highlight certain differences in the proportion of TUNEL-positive cells observed here to the phenotypic outcome (tumor volume) seen between Vexo-AAV6-*iCasp9*- versus AAV6-*iCasp9*-treated groups (Figure 3). This could possibly be due to a late burst in apoptosis in animals treated with Vexo-AAV6 -*iCasp9* vectors. Thus, a further long-term follow-up (>10 days) may be required to assess the complete impact of suicide gene transfer with AAV6 vexosomes.

## Discussion

Exosomes are small extracellular vesicles that are secreted from a cell for communication^32^, ^33^, ^34^ and are known to maintain tissue homeostasis.^35^^,^^36^ Exosomes are secreted out from the cell after the fusion of multivesicular bodies (MVBs) to the cell membrane.^37^ Recently, exosomes have gained attention because of the diversity of the cargo capacity they carry (mRNA, microRNA [miRNA], proteins, DNA)^38^, ^39^, ^40^ and immune-evasion^41^ properties. Several DNA and RNA viruses have been previously shown to be associated with exosomes,^42^, ^43^, ^44^ suggesting a plausible evolutionary conserved mechanism.^45^ Furthermore, the role of exosomes in gene delivery in general^46^ and with vexosomes harvested during AAV production has been explored in different disease settings, such as in hemophilia,^17^ LCA,^19^ or neurological diseases.^47^ The efficacy AAV2- based vexosomes with enhanced transduction of the retinal cells upon intravitreal delivery has been known.^19^ Other studies that have employed vexosomes derived from AAV serotypes, such as AAV1, -6, -7, -8, or -9, have mostly analyzed the rate of formation and distribution of vectors in the cell lysate and culture media.^24^^,^^25^ To the best of our knowledge, this is the first study to demonstrate the therapeutic potential of the Vexo-associated AAV serotype 6 vectors in a murine model of HCC.

HCC is associated with profound mortality and/or morbidity, even after conventional interventions, such as surgical resection or use of tyrosine kinase inhibitors.^48^^,^^49^ We have recently developed a AAV2-based, *iCasp9*-based suicide gene therapy as an alternative approach to treat HCC.^20^ Due to the fact that frequency of high titer neutralizing antibodies to AAV2 in the general population can be as high as ∼70%,^50^ we wished to evaluate an alternate AAV6 serotype for its potential in delivering the *iCasp9* suicide gene. Interestingly, the AAV6 serotype can efficiently target liver cells, including Huh7 cells.^51^^,^^52^ Furthermore, the feasibility and attractiveness of harvesting vexosome-associated AAV6 vectors from spent media, which otherwise are routinely discarded during vector production, motivated us to test the utility of this system for therapeutic suicide gene therapy. It must be highlighted that the vexosomes for our study were isolated from routine packaging conditions of AAV generation without incorporating additional modifications, such as use of exosome-specific media (2% bovine exosome-free fetal bovine serum [FBS]), as described earlier.^16^ Our data demonstrate that AAV6 vexosomes thus generated from routine AAV packaging had a significant therapeutic efficacy when a combination of AAV6-*iCasp9* suicide gene-containing vexosomes and AP20187 was tested (Figure 3). However, our data also showed that the efficacy of Vexo-associated-AAV6 was similar to that of AAV6 naked vectors. Previous studies with AAV serotypes 1, 8, and 9, carrying a bi-bicistronic expression cassette consisting of the human serum protein α1 antitrypsin (AAT) and green fluorescent protein (GFP), demonstrated equivalent protein expression after administration of vector preparations from cell lysates and culture fluids for all three serotypes.^25^ These data highlight that the efficiency of vexosome-based gene therapy can be disease and context specific. It further stands to reason that the efficacy of the vexosomes generated will be dependent on the source and type of packaging cells (human versus insect cells),^53^ physical parameters of vector packaging (media pH, composition, etc.), AAV serotype employed,^13^ and size difference in the expression cassettes packaged.^26^ All of these factors imply a recombinant vector-dependent processing mechanism that determines the AAV/vexosome distribution in media and lysate. Further detailed mechanistic studies are required to understand their impact in generating AAV6 vexosomes.

In addition, previous reports with vexosomes derived from other AAV serotypes, such as AAV8, have known to confer phenotypic correction in hemophilia mice and an increased resistance to neutralizing anti-AAV antibodies.^17^ This is likely due to the fact that Vexo-AAV vectors are naturally enveloped with exosomal membranes and thus, possibly have a propensity to shield the viral vectors from neutralizing antibodies. In the case of the AAV6 vexosomes, a similar outcome may be expected in the setting of neutralizing antibody positivity, but further evaluation of this phenomenon is required in suitable murine models *in vivo*.

In conclusion, we have demonstrated the utility of AAV6-based vexosomal vectors at considerably low doses (2 × 10^10^ vgs) for suicide gene therapy of HCC. The attractiveness of our approach is deriving these highly efficient Vexo-AAV6 vectors from media discarded during routine AAV packaging. Apart from the ease of harvesting these vexosomes, the overall protocol for vexosome isolation is completed in ∼2.5 h, as described earlier,^19^ adding significantly to the appeal of this mode of gene delivery. This approach can be also potentially applicable for the treatment of other solid malignancies, such as breast or head/neck cancer. However, concerted efforts are needed to understand the safety and immunogenicity of vexosomes in multiple settings and further standardize the methods to scale up the production of vexosomes for clinical use.

## Materials and Methods

### Cell Culture

Human AAV293 packaging cells were purchased from Stratagene (San Diego, CA, USA) and the human hepatocellular carcinoma (Huh7) cell line was a kind gift from Dr. Saumitra Das (Indian Institute of Science, Bangalore). The cells were cultured in Iscove’s modified Dulbecco’s medium (IMDM), supplemented with 10% fetal bovine serum (Gibco, Carlsbad, CA, USA), ciprofloxacin (HiMedia Laboratories, Mumbai, India), and piperacillin (MP Biomedicals, Irvine, CA, USA) at 10 μg/mL in a humidified atmosphere, supplemented with 5% CO_2_ at 37°C.

### Vector Preparation

Conventional AAV vectors were generated as described previously.^10^ Briefly, AAV293 cells, expanded in 15 cm^2^ dishes (n = 40), were cotransfected using polyethylenimine (PEI; Polysciences, Warrington, PA, USA) with an equimolar concentration of plasmids carrying the *rep/cap* plasmid (p.AAVR2/C6), an inducible caspase 9 transgene (p.AAV-CBa-*iCasp9*)^20^ and adenoviral helper plasmids (p.Helper). Transfected cultures were maintained in IMDM, supplemented with 10% FBS. About 68 h after transfection, cells were collected by centrifuging the suspension at 1,000 *g* for 5 min. After three freeze-thaw cycles (–80°C, 37°C), AAV particles were purified from the cell lysates by iodixanol gradient centrifugation. The gradient fraction containing AAV vectors was further purified by column chromatography (HiTrap Q column; GE Healthcare Life Sciences, Chicago, IL, USA). Purified vectors were finally concentrated using Amicon Ultra-15 centrifugal filtration devices (Millipore, Bedford, MA, USA). The supernatant culture medium from the same 40, 15 cm plates (800 mL), 68 h post-transfection, was pooled. This conditioned medium fraction was transferred into 50 mL tubes and stored at −80°C for vexosome isolation.

### Vexosome Isolation

Vexosomes were collected from the conditioned media (800 mL) of AAV293 cells cultured for 68 h. The vexosomes were purified by differential centrifugation of the conditioned media, as described previously.^13^^,^^25^ The supernatant media were first centrifuged at 800 *g* for 10 min to sediment the cells and centrifuged at 20,000 *g* for 1 h (Optima L-100K Ultra Centrifuge; Beckman Coulter, Brea, CA, USA) to remove the cellular debris. Vexosomes were separated from the supernatant by further centrifugation at 100,000 *g* for 1.5 h. The pellet was then resuspended in 100 μL of PBS (vexosome fraction).

### Quantitative Polymerase Chain Reaction

The AAV genome titers in both the standard AAV6 and vexosome preparation were measured by a quantitative real-time PCR, as described earlier.^54^ Samples were treated with DNase before quantitative real-time PCR in order to eliminate nonencapsidated DNA. The primers were targeted to the polyadenylation (PolyA) signal in the encapsidated genome, and the quantitative real-time PCR performed on a CFX96 real-time PCR instrument (Bio-Rad, Hercules, CA, USA) using SYBR Green (Promega, Madison, WI, USA). The titers were generated from two replicate analyses and are represented as vgs per milliliter.

### Transmission Electron Microscopy

To characterize the vexosomes, ∼10 μL of either neat or diluted (1:2) vexosomes was adsorbed onto TEM copper grids (Ted Pella, Redding, CA, USA), stained (2% uranyl acetate). The TEM images were acquired in a transmission electron microscope (FEI Technai G^2^, Hillsboro, OR, USA). About 10–20 images of vexosomes from each grid were acquired and further quantified using ImageJ analysis (National Institutes of Health, Bethesda, MD, USA).

### Immunoblotting

The vexosome pellet containing AAV vectors was isolated, as described above, and their protein fraction isolated by using radioimmunoprecipitation assay (RIPA) buffer (Pierce, Thermo Fisher Scientific, Waltham, MA, USA). The protein concentration in the isolated samples was determined by the bicinchoninic acid (BCA) assay (Thermo Fisher Scientific), as per the manufacturer’s instructions. Subsequently, an equal amount of vexosomal protein (25–60 μg) was denatured with a 4× denaturation buffer (Bio-Rad) at 95°C for 10 min. The denatured samples were then separated in a 10% SDS-polyacrylamide gel by electrophoresis. The resolved proteins were transferred onto a polyvinylidene fluoride (PVDF) membrane and further blocked in 2% skimmed milk to minimize nonspecific binding of antibodies. Immunoblotting was performed using anti-human CD9 antibody (Santa Cruz Biotechnology, Dallas, TX, USA), anti-human CD63 antibody (Abcam, Cambridge, MA, USA), or AAV capsid-specific anti-AAV(B1) antibody (Fitzgerald, North Acton, MA, USA). The chemiluminescent signals were developed using the West PicoPlus enhanced chemiluminescence (ECL) substrate kit (Thermo Fisher Scientific) and captured using a ChemiDoc Imager (ECL; Thermo Fisher Scientific).

### AAV6 Vexosome-Mediated Cytotoxicity Assays *In Vitro*

About ∼1.5 × 10^4^ Huh7 cells per well in a 48-well plate were either mock (PBS) infected or infected (MOI: 5 × 10^4^ vgs/cell) with AAV6-*iCasp9* vectors or AAV6-*iCasp9* vexosomes. 24 h postinfection, transfected cells were treated with AP20187 (10 nm; Ariad Pharmaceuticals, Cambridge, MA, USA). 2 days later, we measured the cell viability by an ATP assay, per the commercial protocol (CellTiter-Glo; Promega) and as previously reported.^20^

### AAV6 Vexosome-Mediated Suicide Gene Delivery *In Vivo*

Our animal studies utilized athymic nude mice (8–10 weeks old) from a BALB/c genetic background (National Institute of Nutrition, Hyderabad, India). The animal use and experimentation were approved by the Institutional Animal Ethics Committee (IIT-Kanpur, India). For xenotransplantation, we subcutaneously administered ∼5 million Huh7 cells in a mixture of 25% Matrigel (Sigma-Aldrich, St. Louis, MO, USA) in 200 μL vol. When the animals developed palpable tumors (150–200 mm^3^ size), they were randomized into three groups: mock, AAV6-*iCasp9*, and vexosomal fraction (Vexo-AAV6-*iCasp9*). Animals from the treatment group received ∼2 × 10^10^ vgs of each of the vectors intratumorally. Subsequently, vector-injected animals were administered with AP20187 thrice and 2 days apart from each dose of 1 mg/kg body weight, as described earlier.^20^ The animals (n = 7-10 per group) were continuously evaluated and the tumor volumes calculated (0.5 × L [largest diameter] × W^2^ [shortest diameter in millimeters]), as described previously.^55^ At the end of experiment, animals were euthanized humanely and the tumor samples harvested, as previously described.^20^

### Histology and *In Situ* TUNEL Assays

Tumors from each group were harvested, washed briefly with PBS, fixed, and cryosectioned (6 μm). The sections (n = 3 per animal; 3 animals per group) were then stained with hematoxylin and eosin. We also performed an *in situ* TUNEL, and counterstaining with 4′, 6-diamidino-2-phenylindole (DAPI; Thermo Fisher Scientific) was performed to detect apoptotic cells, according to the manufacturer’s protocol (Invitrogen). Images were captured in a Leica DM5000B microscope (Leica Microsystems, Wetzlar, Germany).

### Statistical Analysis

All values are expressed as mean ± standard deviation (SD). Statistical analysis was performed using analysis of variance (ANOVA) tests using GraphPad Prism 7.0 software (GraphPad Software, La Jolla, CA). A p value of <0.05 between the control and test groups was considered to be statistically significant.

## Author Contributions

N.K., S.M., and S.B. conducted the experiments. G.R.J. supervised the study. N.K. and G.R.J. wrote the manuscript.

## Conflicts of Interest

The authors declare no competing interests.

## References

[1] Rolling F., Samulski R.J. (1995). AAV as a viral vector for human gene therapy. Generation of recombinant virus. Mol. Biotechnol..

[2] Srivastava A. (2016). In vivo tissue-tropism of adeno-associated viral vectors. Curr. Opin. Virol..

[3] Nathwani A.C., Tuddenham E.G., Rangarajan S., Rosales C., McIntosh J., Linch D.C., Chowdary P., Riddell A., Pie A.J., Harrington C. (2011). Adenovirus-associated virus vector-mediated gene transfer in hemophilia B. N. Engl. J. Med..

[4] Bainbridge J.W., Smith A.J., Barker S.S., Robbie S., Henderson R., Balaggan K., Viswanathan A., Holder G.E., Stockman A., Tyler N. (2008). Effect of gene therapy on visual function in Leber’s congenital amaurosis. N. Engl. J. Med..

[5] Hauswirth W.W., Aleman T.S., Kaushal S., Cideciyan A.V., Schwartz S.B., Wang L., Conlon T.J., Boye S.L., Flotte T.R., Byrne B.J., Jacobson S.G. (2008). Treatment of leber congenital amaurosis due to RPE65 mutations by ocular subretinal injection of adeno-associated virus gene vector: short-term results of a phase I trial. Hum. Gene Ther..

[6] Nathwani A.C., Reiss U.M., Tuddenham E.G., Rosales C., Chowdary P., McIntosh J., Della Peruta M., Lheriteau E., Patel N., Raj D. (2014). Long-term safety and efficacy of factor IX gene therapy in hemophilia B. N. Engl. J. Med..

[7] Mingozzi F., High K.A. (2011). Therapeutic in vivo gene transfer for genetic disease using AAV: progress and challenges. Nat. Rev. Genet..

[8] Yuan Z., Qiao C., Hu P., Li J., Xiao X. (2011). A versatile adeno-associated virus vector producer cell line method for scalable vector production of different serotypes. Hum. Gene Ther..

[9] Clark K.R. (2002). Recent advances in recombinant adeno-associated virus vector production. Kidney Int..

[10] Mary B., Maurya S., Arumugam S., Kumar V., Jayandharan G.R. (2019). Post-translational modifications in capsid proteins of recombinant adeno-associated virus (AAV) 1-rh10 serotypes. FEBS J..

[11] Potter M., Lins B., Mietzsch M., Heilbronn R., Van Vliet K., Chipman P., Agbandje-McKenna M., Cleaver B.D., Clément N., Byrne B.J., Zolotukhin S. (2014). A simplified purification protocol for recombinant adeno-associated virus vectors. Mol. Ther. Methods Clin. Dev..

[12] Gao G., Qu G., Burnham M.S., Huang J., Chirmule N., Joshi B., Yu Q.C., Marsh J.A., Conceicao C.M., Wilson J.M. (2000). Purification of recombinant adeno-associated virus vectors by column chromatography and its performance in vivo. Hum. Gene Ther..

[13] Vandenberghe L.H., Xiao R., Lock M., Lin J., Korn M., Wilson J.M. (2010). Efficient serotype-dependent release of functional vector into the culture medium during adeno-associated virus manufacturing. Hum. Gene Ther..

[14] Maguire C.A., Balaj L., Sivaraman S., Crommentuijn M.H., Ericsson M., Mincheva-Nilsson L., Baranov V., Gianni D., Tannous B.A., Sena-Esteves M. (2012). Microvesicle-associated AAV vector as a novel gene delivery system. Mol. Ther..

[15] Hudry E., Martin C., Gandhi S., György B., Scheffer D.I., Mu D., Merkel S.F., Mingozzi F., Fitzpatrick Z., Dimant H. (2016). Exosome-associated AAV vector as a robust and convenient neuroscience tool. Gene Ther..

[16] György B., Fitzpatrick Z., Crommentuijn M.H., Mu D., Maguire C.A. (2014). Naturally enveloped AAV vectors for shielding neutralizing antibodies and robust gene delivery in vivo. Biomaterials.

[17] Meliani A., Boisgerault F., Fitzpatrick Z., Marmier S., Leborgne C., Collaud F., Simon Sola M., Charles S., Ronzitti G., Vignaud A. (2017). Enhanced liver gene transfer and evasion of preexisting humoral immunity with exosome-enveloped AAV vectors. Blood Adv..

[18] György B., Sage C., Indzhykulian A.A., Scheffer D.I., Brisson A.R., Tan S., Wu X., Volak A., Mu D., Tamvakologos P.I. (2017). Rescue of Hearing by Gene Delivery to Inner-Ear Hair Cells Using Exosome-Associated AAV. Mol. Ther..

[19] Wassmer S.J., Carvalho L.S., György B., Vandenberghe L.H., Maguire C.A. (2017). Exosome-associated AAV2 vector mediates robust gene delivery into the murine retina upon intravitreal injection. Sci. Rep..

[20] Khan N., Bammidi S., Chattopadhyay S., Jayandharan G.R. (2019). Combination Suicide Gene Delivery with an Adeno-Associated Virus Vector Encoding Inducible Caspase-9 and a Chemical Inducer of Dimerization Is Effective in a Xenotransplantation Model of Hepatocellular Carcinoma. Bioconjug. Chem..

[21] Patel G.K., Khan M.A., Zubair H., Srivastava S.K., Khushman M., Singh S., Singh A.P. (2019). Comparative analysis of exosome isolation methods using culture supernatant for optimum yield, purity and downstream applications. Sci. Rep..

[22] Andreu Z., Yáñez-Mó M. (2014). Tetraspanins in extracellular vesicle formation and function. Front. Immunol..

[23] Schiller L.T., Lemus-Diaz N., Rinaldi Ferreira R., Böker K.O., Gruber J. (2018). Enhanced Production of Exosome-Associated AAV by Overexpression of the Tetraspanin CD9. Mol. Ther. Methods Clin. Dev..

[24] Okada T., Nonaka-Sarukawa M., Uchibori R., Kinoshita K., Hayashita-Kinoh H., Nitahara-Kasahara Y., Takeda S., Ozawa K. (2009). Scalable purification of adeno-associated virus serotype 1 (AAV1) and AAV8 vectors, using dual ion-exchange adsorptive membranes. Hum. Gene Ther..

[25] Lock M., Alvira M., Vandenberghe L.H., Samanta A., Toelen J., Debyser Z., Wilson J.M. (2010). Rapid, simple, and versatile manufacturing of recombinant adeno-associated viral vectors at scale. Hum. Gene Ther..

[26] Piras B.A., Drury J.E., Morton C.L., Spence Y., Lockey T.D., Nathwani A.C., Davidoff A.M., Meagher M.M. (2016). Distribution of AAV8 particles in cell lysates and culture media changes with time and is dependent on the recombinant vector. Mol. Ther. Methods Clin. Dev..

[27] Yagyu S., Hoyos V., Del Bufalo F., Brenner M.K. (2015). An Inducible Caspase-9 Suicide Gene to Improve the Safety of Therapy Using Human Induced Pluripotent Stem Cells. Mol. Ther..

[28] Sápi J., Kovács L., Drexler D.A., Kocsis P., Gajári D., Sápi Z. (2015). Tumor Volume Estimation and Quasi-Continuous Administration for Most Effective Bevacizumab Therapy. PLoS ONE.

[29] Singh S.S., Mehedint D.C., Ford O.H., Jeyaraj D.A., Pop E.A., Maygarden S.J., Ivanova A., Chandrasekhar R., Wilding G.E., Mohler J.L. (2009). Comparison of ACINUS, caspase-3, and TUNEL as apoptotic markers in determination of tumor growth rates of clinically localized prostate cancer using image analysis. Prostate.

[30] Duan W.R., Garner D.S., Williams S.D., Funckes-Shippy C.L., Spath I.S., Blomme E.A. (2003). Comparison of immunohistochemistry for activated caspase-3 and cleaved cytokeratin 18 with the TUNEL method for quantification of apoptosis in histological sections of PC-3 subcutaneous xenografts. J. Pathol..

[31] Wieder R. (2005). TUNEL assay as a measure of chemotherapy-induced apoptosis. Methods Mol. Med..

[32] Mathivanan S., Ji H., Simpson R.J. (2010). Exosomes: extracellular organelles important in intercellular communication. J. Proteomics.

[33] Record M., Carayon K., Poirot M., Silvente-Poirot S. (2014). Exosomes as new vesicular lipid transporters involved in cell-cell communication and various pathophysiologies. Biochim. Biophys. Acta.

[34] Raposo G., Stoorvogel W. (2013). Extracellular vesicles: exosomes, microvesicles, and friends. J. Cell Biol..

[35] Baixauli F., López-Otín C., Mittelbrunn M. (2014). Exosomes and autophagy: coordinated mechanisms for the maintenance of cellular fitness. Front. Immunol..

[36] Hessvik N.P., Øverbye A., Brech A., Torgersen M.L., Jakobsen I.S., Sandvig K., Llorente A. (2016). PIKfyve inhibition increases exosome release and induces secretory autophagy. Cell. Mol. Life Sci..

[37] Harding C., Heuser J., Stahl P. (1983). Receptor-mediated endocytosis of transferrin and recycling of the transferrin receptor in rat reticulocytes. J. Cell Biol..

[38] Mittelbrunn M., Gutiérrez-Vázquez C., Villarroya-Beltri C., González S., Sánchez-Cabo F., González M.A., Bernad A., Sánchez-Madrid F. (2011). Unidirectional transfer of microRNA-loaded exosomes from T cells to antigen-presenting cells. Nat. Commun..

[39] Nolte-’t Hoen E.N.M., Buermans H.P.J., Waasdorp M., Stoorvogel W., Wauben M.H.M., ’t Hoen P.A. (2012). Deep sequencing of RNA from immune cell-derived vesicles uncovers the selective incorporation of small non-coding RNA biotypes with potential regulatory functions. Nucleic Acids Res..

[40] Pigati L., Yaddanapudi S.C., Iyengar R., Kim D.J., Hearn S.A., Danforth D., Hastings M.L., Duelli D.M. (2010). Selective release of microRNA species from normal and malignant mammary epithelial cells. PLoS ONE.

[41] Kouwaki T., Okamoto M., Tsukamoto H., Fukushima Y., Oshiumi H. (2017). Extracellular Vesicles Deliver Host and Virus RNA and Regulate Innate Immune Response. Int. J. Mol. Sci..

[42] Feng Z., Hensley L., McKnight K.L., Hu F., Madden V., Ping L., Jeong S.H., Walker C., Lanford R.E., Lemon S.M. (2013). A pathogenic picornavirus acquires an envelope by hijacking cellular membranes. Nature.

[43] Yang Y., Han Q., Hou Z., Zhang C., Tian Z., Zhang J. (2017). Exosomes mediate hepatitis B virus (HBV) transmission and NK-cell dysfunction. Cell. Mol. Immunol..

[44] Wiley R.D., Gummuluru S. (2006). Immature dendritic cell-derived exosomes can mediate HIV-1 trans infection. Proc. Natl. Acad. Sci. USA.

[45] Masciopinto F., Giovani C., Campagnoli S., Galli-Stampino L., Colombatto P., Brunetto M., Yen T.S., Houghton M., Pileri P., Abrignani S. (2004). Association of hepatitis C virus envelope proteins with exosomes. Eur. J. Immunol..

[46] Jiang X.C., Gao J.Q. (2017). Exosomes as novel bio-carriers for gene and drug delivery. Int. J. Pharm..

[47] Orefice N.S., Souchet B., Braudeau J., Alves S., Piguet F., Collaud F., Ronzitti G., Tada S., Hantraye P., Mingozzi F. (2019). Real-Time Monitoring of Exosome Enveloped-AAV Spreading by Endomicroscopy Approach: A New Tool for Gene Delivery in the Brain. Mol. Ther. Methods Clin. Dev..

[48] Forner A., Llovet J.M., Bruix J. (2012). Hepatocellular carcinoma. Lancet.

[49] Cheng A.L., Kang Y.K., Chen Z., Tsao C.J., Qin S., Kim J.S., Luo R., Feng J., Ye S., Yang T.S. (2009). Efficacy and safety of sorafenib in patients in the Asia-Pacific region with advanced hepatocellular carcinoma: a phase III randomised, double-blind, placebo-controlled trial. Lancet Oncol..

[50] Calcedo R., Vandenberghe L.H., Gao G., Lin J., Wilson J.M. (2009). Worldwide epidemiology of neutralizing antibodies to adeno-associated viruses. J. Infect. Dis..

[51] Sayroo R., Nolasco D., Yin Z., Colon-Cortes Y., Pandya M., Ling C., Aslanidi G. (2016). Development of novel AAV serotype 6 based vectors with selective tropism for human cancer cells. Gene Ther..

[52] Rezvani M., Español-Suñer R., Malato Y., Dumont L., Grimm A.A., Kienle E., Bindman J.G., Wiedtke E., Hsu B.Y., Naqvi S.J. (2016). In Vivo Hepatic Reprogramming of Myofibroblasts with AAV Vectors as a Therapeutic Strategy for Liver Fibrosis. Cell Stem Cell.

[53] Urabe M., Ding C., Kotin R.M. (2002). Insect cells as a factory to produce adeno-associated virus type 2 vectors. Hum. Gene Ther..

[54] Aurnhammer C., Haase M., Muether N., Hausl M., Rauschhuber C., Huber I., Nitschko H., Busch U., Sing A., Ehrhardt A., Baiker A. (2012). Universal real-time PCR for the detection and quantification of adeno-associated virus serotype 2-derived inverted terminal repeat sequences. Hum. Gene Ther. Methods.

[55] Tomayko M.M., Reynolds C.P. (1989). Determination of subcutaneous tumor size in athymic (nude) mice. Cancer Chemother. Pharmacol..

